# The Japanese Breast Cancer Society clinical practice guidelines for surgical treatment of breast cancer, 2018 edition

**DOI:** 10.1007/s12282-019-01030-w

**Published:** 2019-12-12

**Authors:** Masafumi Inokuchi, Goro Kutomi, Yuko Kijima, Takehiko Sakai, Masataka Sawaki, Tadahiko Shien, Noriko Hanamura, Kenji Yano, Noriaki Wada, Shigehira Saji, Hiroji Iwata

**Affiliations:** 1 Department of Breast Endocrine Surgery, Kanazawa Medical University, 1-1, Uchinada-Daigaku, Kahoku, Ishikawa 920-0293 Japan; 2 Department of Surgery, Surgical Oncology and Science, Sapporo Medical University Hospital, Sapporo, Hokkaido Japan; 3 Department of Breast Surgery, School of Medicine, Fujita Health University, Toyoake, Aichi Japan; 4 Breast Oncology Center, Cancer Institute Hospital of the Japanese Foundation for Cancer Research, Tokyo, Japan; 5 Department of Breast Oncology, Aichi Cancer Center Hospital, Nagoya, Aichi Japan; 6 Department of Breast and Endocrine Surgery, Okayama University Hospital, Okayama, Japan; 7 Breast Center, Saiseikai Matsusaka General Hospital, Matsuzaka, Mie Japan; 8 Department of Plastic Surgery, Osaka Breast Clinic, Osaka, Japan; 9 Department of General and Breast Surgery, Tokyo Dental College Ichikawa General Hospital, Ichikawa, Chiba Japan; 10 Department of Medical Oncology, Fukushima Medical University, Fukushima, Japan

**Keywords:** Clinical practice guideline, Clinical question, Surgical treatment of breast cancer

## Abstract

We have prepared the Japanese Breast Cancer Society clinical practice guidelines (CPGs) for surgical treatment of breast cancer, 2018 update after a systematic review (SR) of the literature based upon the Medical Information Network Distribution Service (Minds) procedure. The CPG committee for surgical treatment of breast cancer, composed of breast surgeons and plastic surgeons treating breast cancer, has developed the CPGs. Eight clinical questions (CQs) were selected and divided roughly into the following five categories: (1) breast surgery in initial therapy (CQs 1–3); (2) axillary surgery in initial therapy (CQs 4–5); (3) breast reconstruction in initial therapy (CQ 6); (4) surgical treatment for recurrent and metastatic breast cancer (CQs 7–8); and (5) others. Recommendations for these CQs were decided by the GRADE grid method. In addition, 4 outlines, 14 background questions (BQs), and 12 future research questions (FQs) were also selected. Statements for these BQs and FQs are provided. We developed the updated CPGs for surgical treatment of breast cancer, 2018, which include 8 CQs and recommendations. As a decision-making tool for the understanding and treatment of breast cancer, these guidelines will help surgical oncologists dealing with breast cancer, medical staff, and patients, along with their family members.

## Introduction

In the Breast Cancer Practice Guidelines, 2018 edition, guidelines were developed while weighing the balance of benefits and harms, and the Medical Information Network Distribution Service (Minds) Handbook for Clinical Practice Guideline Development 2014 [1] was used as a reference. In contrast to the method of the previous guidelines, we conducted a systematic review (SR) for each clinical question (CQ) and decided on the recommendations. In preparing clinical practice guidelines (CPGs), the CPG subcommittee for surgical treatment of breast cancer, composed of breast surgeons and plastic surgeons treating breast cancer, worked over a period of about one and a half years.

Initially, we created scopes in five categories that were significant clinical problems in surgery: (1) breast surgery in initial therapy; (2) axillary surgery in initial therapy; (3) breast reconstruction in initial therapy; (4) surgical treatment for recurrent and metastatic breast cancer; and (5) others. While further checking the CQs of the last, 2015 edition [2], we developed the 9 CQs for this 2018 edition. The process for CQs was: (1) setting of outcomes; (2) collecting evidence; (3) primary screening; (4) secondary screening; (5) evaluation of each article; (6) evaluation of the strength of the evidence; (7) integration of the evidence; (8) creation of recommendations; (9) recommendation decision meeting; (10) writing of the commentary; (11) evaluation by the Evaluation Committee; and (12) board approval and its final decision.

Furthermore, we created background questions (BQs) and future research questions (FQs). Background questions (BQs) were positioned as standard treatments in previous guidelines for each important clinical subject. Future research questions (FQs), which are currently insufficiently researched but may be taken up as CQs in the future, were also organized and set up among the CQs of the 2015 edition [2] with added statements and commentary. Table 1 shows the structure of the guidelines for surgical treatment.

**Table 1:** Scope and structure of the guidelines for surgical treatment

(1) Breast surgery in initial therapy	Outline, BQ 1–3, CQ 1–3, FQ 1
(2) Axillary surgery in initial therapy	Outline, BQ 4–9, CQ 4–5, FQ 2–4
(3) Breast reconstruction in initial therapy	Outline, BQ 10, CQ 6, FQ 5
(4) Surgical treatment for recurrent and metastatic breast cancer	Outline, CQ7–8, FQ 6–10
(5) Others	BQ 11–14, FQ 11–12

*BQ* background question, *CQ* clinical question, *FQ* future research question

For this edition, we developed 8 CQs and 14 BQs. There is one new CQ (CQ1), and we selected the other seven CQs from the CQs of the 2015 edition. In this article, we introduce the contents of the CQs and BQs.

## Clinical questions and recommendations

* Structure: CQ sentence, recommendation, strength of recommendation (Table 2 shows recommendation grades.), and strength of evidence. (Table 3 shows strength of evidence.)

**Table 2:** Recommendation grade

Strength of recommendation	Statement	Clinical meaning
1	Strongly recommend to do	It is possible to paraphrase you should examine
2	Weekly recommend to do	If anything recommends to do based on considering balance between harm and benefit, patient’s values
3	Weekly recommend to not to do	If anything recommends to not to do based on considering balance between harm and benefit, patient’s values
4	Strongly recommend to not to do	You should not examine because of CQ which extremely exceed harm than benefit

**Table 3:** Strength of evidence

Strength	Example
Strong	Several high-quality studies with consistent results
Moderate	One high-quality study
Weak	Studies with severe limitations
Very weak	Studies with very severe limitations or expert opinion

### Breast surgery in the initial treatment of breast cancer

**CQ1: Is surgery recommended for patients with non-invasive ductal cancer (ductal carcinoma in situ: DCIS)?**

(Recommendation) Surgery for patients with non-invasive ductal carcinoma is weakly recommended. [Strength of recommendation (SoR): 3, strength of evidence (SoE): very weak]

**CQ2: In breast-conserving surgery (BCS) for invasive/non-invasive ductal carcinoma, is additional surgery recommended for patients with positive surgical margins?**

(Recommendation) Additional surgery is weakly recommended for patients with positive surgical margins in BCS. (SoR: 2, SoE: weak)

**CQ3: Is skin-sparing or nipple/areola-sparing mastectomy recommended in breast reconstruction?**

**CQ3a: Is skin-sparing mastectomy (SSM) recommended?**

(Recommendation) Skin-sparing mastectomy is weakly recommended. (SoR: 2, SoE: weak)

**CQ3b: Is nipple-sparing mastectomy (NSM) recommended?**

(Recommendation) Nipple-sparing mastectomy is weakly recommended. (SoR: 2, SoE: weak)

### Axillary surgery in the initial treatment of breast cancer

**CQ4: Is no further axillary surgery for patients with sentinel node metastasis recommended?**

**CQ4a: In cases of micrometastases**

(Recommendation) Is no further axillary surgery is strongly recommended. (SoR: 1, SoE: moderate)

**CQ4b: In cases of macrometastases**

**CQ4b-1: Breast-conserving therapy**

(Recommendation) Is no further axillary surgery is weakly recommended. (SoR: 2, SoE: moderate)

**CQ4b-2: Total mastectomy (without radiation therapy)**

(Recommendation) Further axillary surgery is strongly recommended. (SoR: 4, SoE: very weak)

**CQ4b-3: Total mastectomy (with radiation therapy)**

(Recommendation) Is no further axillary surgery is weakly recommended. (SoR: 2, SoE: weak)

**CQ5: Is sentinel lymph node biopsy (SLNB) after neoadjuvant chemotherapy (NAC) recommended for avoidance of ALND?**

**CQ5a: Is SLNB for clinical node-negative breast cancer before and after NAC recommended?**

(Recommendation) SLNB is weakly recommended. (SoR: 2, SoE: weak)

**CQ5b: Is SLNB recommended, if an ipsilateral axillary lymph node is initially positive, and it is clinically negative after NAC?**

(Recommendation) It is weakly recommended not to perform SLNB (recommended to perform ALND) (SoR: 3, SoE: weak)

### Breast reconstruction in the initial treatment of breast cancer

**CQ6: Is immediate breast reconstruction recommended after total mastectomy for clinically node-positive breast cancer patients who desire breast reconstruction?**

(Recommendation) Immediate breast reconstruction after total mastectomy is weakly recommended for breast cancer patients with node-positive breast cancer who desire breast reconstruction. (SoR: 2, SoE: weak)

### Surgery for metastatic and recurrent breast cancer

**CQ7: Is primary tumor resection to improve prognosis recommended for Stage IV breast cancer?**

(Recommendation) It is weakly recommended not to perform primary tumor resection to improve prognosis for Stage IV breast cancer. (SoR: 3, SoE: moderate)

**CQ8: Is surgical resection recommended for regional lymph node recurrence?**

**CQ8a: For ipsilateral axillary lymph node recurrence after ALND**

(Recommendation) Surgical resection is weakly recommended. (SoR: 2, SoE: very weak)

**CQ8b: For ipsilateral supraclavicular lymph node recurrence**

(Recommendation) It is weakly recommended not to perform surgical resection. (SoR: 3, SoE: very weak)

## Background questions and statements

* Structure: BQ sentence and statement.

### Breast surgery in the initial treatment of breast cancer

**BQ1: Is breast-conserving therapy (BCT) recommended for ductal carcinoma in situ (DCIS)?**

(Statement) BCT for DCIS is performed according to the indication for BCT for invasive breast cancer.

**BQ2: Is BCT recommended as a local therapy for stage I and II invasive breast cancer?**

(Statement) As a local treatment for stage I and II breast cancer, BCT is regarded as the first choice if indicated, because it provides a survival equivalent to mastectomy.

**BQ3: Is BCT recommended for invasive breast cancer patients responding to neoadjuvant chemotherapy (NAC)?**

(Statement) BCT is recommended for invasive breast cancer patients responding to NAC.

### Axillary surgery in the initial treatment of breast cancer

**BQ4: Is level I and II ALND recommended for patients with clinically positive axillary lymph nodes?**

(Statement) Level I and II ALND is recommended for patients with clinically positive axillary lymph nodes.

**BQ5: Is no further axillary surgery for clinically node-negative breast cancer patients without SLN metastases recommended?**

(Statement) No further axillary surgery for women with clinically axillary node-negative breast cancer who do not have nodal metastases by SLNB is the standard treatment.

**BQ6: Is SLNB with a combination of blue dye and radioisotope (RI) recommended?**

(Statement) The combination of blue dye and RI showed slightly better identification rates than either method used alone; therefore the combined method is standard, but the single methods are also acceptable.

**BQ7: Is SLNB recommended for patients with an initial diagnosis of DCIS?**

(Statements) SLNB should not be performed for patients for scheduled breast-conserving therapy when the postoperative pathological diagnosis is considered to be DCIS.

∙ If it is difficult to perform SLNB at a later time, SLNB together with primary tumor resection is acceptable.

**BQ8: Is upper limb rehabilitation recommended after ALND?**

(Statement) Upper limb rehabilitation should be performed after ALND.

**BQ9: Is prevention and treatment of ipsilateral postoperative upper limb lymphedema for breast cancer patients recommended?**

(Statements)

① Prevention:

Appropriate preventive education (skin care) and exercise therapy for postoperative lymphedema reduce the incidence rate and increase the opportunity for early intervention at onset. There are no data that clearly show the preventive effects of manual self-lymph drainage performed by patients and families on upper limb lymphedema.

② Treatment:

Combined treatment based on compression therapy using elastic garments and bandages and exercise under compression is effective regardless of the stage of upper limb lymphedema.

### Breast reconstruction in the initial treatment of breast cancer

**BQ10: Is breast reconstruction recommended for patients with a history of chest wall irradiation?**

(Statement) Breast implant-based reconstruction is not encouraged.

Breast reconstruction using autologous tissue is possible.

### Others

**BQ11: Is surgery recommended for pregnant patients with breast cancer?**

(Statement) Surgery is recommended even for pregnant patients with breast cancer.

**BQ12: Is surgery recommended for elderly women with breast cancer?**

(Statement) Surgery is also the standard treatment for elderly women with breast cancer if they are healthy enough to tolerate surgery.

**BQ13: Is routine use of antibiotic prophylaxis for breast cancer surgery recommended?**

(Statement) Prophylactic antibiotics should be given during breast cancer surgery.

**BQ14: Is surgery recommended for phyllodes tumor?**

(Statements) The standard treatment for patients with a pathological diagnosis of phyllodes tumor is complete surgical resection. Taking into account the size and grade of the tumor, it can be necessary to have wide local excision with appropriate margins.

## Conclusion

This time, for eight CQs in the clinical practice guidelines, 2018 edition, SR was performed to determine the recommendations. It is hoped that these guidelines can be used as a tool for physicians and patients in shared decision-making when deciding on breast cancer treatment.
