# Correction to: The Japanese Breast Cancer Society clinical practice guidelines for surgical treatment of breast cancer, 2018 edition

**DOI:** 10.1007/s12282-021-01256-7

**Published:** 2021-05-19

**Authors:** Masafumi Inokuchi, Goro Kutomi, Yuko Kijima, Takehiko Sakai, Masataka Sawaki, Tadahiko Shien, Noriko Hanamura, Kenji Yano, Noriaki Wada, Shigehira Saji, Hiroji Iwata

**Affiliations:** 1 Department of Breast Endocrine Surgery, Kanazawa Medical University, 1-1, Uchinada-Daigaku, Kahoku, Ishikawa 920-0293 Japan; 2 Department of Surgery, Surgical Oncology and Science, Sapporo Medical University Hospital, Sapporo, Hokkaido Japan; 3 Department of Breast Surgery, School of Medicine, Fujita Health University, Toyoake, Aichi Japan; 4 Breast Oncology Center, Cancer Institute Hospital of the Japanese Foundation for Cancer Research, Tokyo, Japan; 5 Department of Breast Oncology, Aichi Cancer Center Hospital, Nagoya, Aichi Japan; 6 Department of Breast and Endocrine Surgery, Okayama University Hospital, Okayama, Japan; 7 Breast Center, Saiseikai Matsusaka General Hospital, Matsuzaka, Mie Japan; 8 Department of Plastic Surgery, Osaka Breast Clinic, Osaka, Japan; 9 Department of General and Breast Surgery, Tokyo Dental College Ichikawa General Hospital, Ichikawa, Chiba Japan; 10 Department of Medical Oncology, Fukushima Medical University, Fukushima, Japan

## Correction to: Breast Cancer (2020) 27: 4–8 10.1007/s12282-019-01030-w

The article “The Japanese Breast Cancer Society clinical practice guidelines for surgical treatment of breast cancer, 2018 edition”, written by Masafumi Inokuchi, Goro Kutomi, Yuko Kijima, Takehiko Sakai, Masataka Sawaki, Tadahiko Shien, Noriko Hanamura, Kenji Yano, Noriaki Wada, Shigehira Saji, Hiroji Iwata, was originally published electronically on the publisher’s internet portal on 12 December 2019 without open access. After publication in volume 27, issue 1, page 4–8, the author decided to opt for Open Choice and to make the article an Open Access publication. Therefore, the copyright of the article has been changed to © The Author(s) and the article is forthwith distributed under the terms of the Creative Commons Attribution 4.0 International License, which permits use, sharing, adaptation, distribution and reproduction in any medium or format, as long as you give appropriate credit to the original author(s) and the source, provide a link to the Creative Commons licence, and indicate if changes were made.

The images or other third party material in this article are included in the article’s Creative Commons licence, unless indicated otherwise in a credit line to the material. If material is not included in the article’s Creative Commons licence and your intended use is not permitted by statutory regulation or exceeds the permitted use, you will need to obtain permission directly from the copyright holder.

To view a copy of this licence, visit http://creativecommons.org/licenses/by/4.0/

The original article has been updated.

